# Effect of Surface Roughness on the Electrical Performances of CPW Transmission Lines Used in Future Ultra-High Frequency Applications

**DOI:** 10.3390/mi14010104

**Published:** 2022-12-30

**Authors:** Zhiqiang Chen, Wenchao Tian

**Affiliations:** School of Mechano-Electronic Engineering, Xidian University, Xi’an 710071, China

**Keywords:** surface roughness, skin depth, CPW transmission line, modified rough coefficient

## Abstract

The development of integrated circuits and packaging technology has led to smaller and smaller transmission line sizes and higher and higher operating frequencies up to nearly 100 GHz. However, the skinning depth of transmission lines due to eddy currents becomes smaller and smaller as the operating frequency of coplanar wave guide (CPW) transmission lines becomes higher and higher, while the reduction of device size makes the skinning depth consistent with the surface roughness of the device. In this paper, the concept of modified roughness coefficient was proposed based on the existing correlation factor. The concept of threshold modified roughness coefficient was proposed with a 20 dB reflection coefficient as the threshold value. The effect of surface roughness on transmission line transmission performance at frequencies above 100 GHz up to 1000 GHz was investigated. It was found that when the operating frequency of the signal was greater than the threshold roughness coefficient, the effect of surface roughness on the transmission line reflection coefficient should be considered. The modified roughness coefficient in this paper could quickly determine the effect of surface roughness on transmission line performance at different frequencies.

## 1. Introduction

Integrated circuits (ICs) and electronic packages continue to evolve toward miniaturization due to the development of technology. In addition, the processing technology of microelectromechanical systems (MEMS) has further intensified this process. At the same time, the transmission speed of electronic products is also increasing, with signal transmission rates approaching or even exceeding 100 GHz [1,2]. However, phenomena such as skin effect at high frequencies are becoming more and more serious, especially when the skin depth is comparable to the coplanar wave guide (CPW) transmission line surface roughness, which can seriously affect the CPW transmission line impedance. These phenomena can lead to degradation of transmission line signal performance and signal integrity issues, especially in ultra-high frequency (UHF), and must therefore be considered during the design process. According to the experimental results in [3], a 10–50% higher loss of rough conductor was obtained compared with smooth conductors.

Some work has been done concerning the surface roughness related problems at multigigahertz. In 1949, Samuel Morgan researched the effect of surface roughness on conductor losses using a 2D surface distortion model [4]. Subsequently, several dedicated geometry models were proposed to describe the surface roughness of the conductor, such as the rectangular shape [5], triangular shape [4,6], hexagonal pyramid [7], hemisphere [8,9], stacked snowball [10,11,12], semi-cylindrical [13], certain arrangements of spheres [14], infinite periodic structures [15], and fractal geometries [16,17,18,19,20]. Although these geometry surface roughness models were easy to understand and simple, they were only suitable for some specific structures. In addition, the filament model [21] and stochastic macromodel [22] were also used to describe the surface roughness problems. During the surface roughness analysis, the root mean square (RMS) of the surface height was mostly used during the modeling [9,23,24,25,26,27,28]. On the other hand, analytical models were also used to study the surface roughness of the conductors, such as the gradient model [29,30,31,32,33,34,35], state-space formulation [33], Green’s function [36,37,38], fast Fourier transformation (FFT) [39], method of momentum (MoM) [40], small perturbation method [24], and Gaussian function from Campbell’s theorem [27,28]. Some other researchers added correction factors to change the smooth surface models to rough surface models [11,41]. This was a fast way to determine the surface roughness effect on high frequency devices. Finally, some methodologies were proposed based on the aforementioned research results [42,43,44]. Levie et al. [6] examined the effect of surface roughness of solid electrodes on electrochemical measurements and presented a model and its mathematical consequences describing the effects in a semi-quantitative way.

In this paper, we proposed a modified roughness coefficient based on the present theory. The proposed modified roughness coefficient could be used to determine the effect of surface roughness on the reflection coefficient at ultra-high frequency. We calculated the critical modified roughness coefficient according to the critical reflection coefficient and maximum frequency of different transmission lines with different surface roughness.

## 2. Theoretical Analysis

### 2.1. Ideal Impedance of the Planar Transmission Line

**Figure 1:**
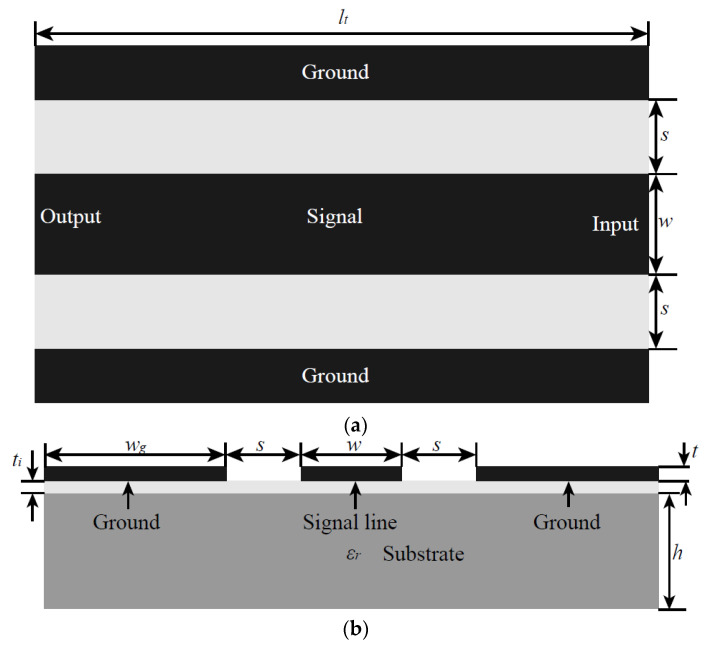
Structure of a transmission line: (**a**) top view; (**b**) section view.

Figure 1 shows the structure of a CPW transmission line in radio frequency (RF). The metal layer was deposited on the substrate layer. Subsequently, the signal and ground lines were patterned on the substrate. The RF signal is transmitted from the RF input of the coplanar waveguide between the ground and signal lines and finally to the RF output. In Figure 1, *s* is the distance between the signal line and ground line, *w* is the width of the signal line, *w_g_* is the width of the ground line, *t* is the thickness of the metal layer, *t_i_* is the think of the insulator layer (insulator layer is typically used in semi-conductor substrate like Si), and *h* is the thickness of the substrate; *ε_r_* is the relative permittivity of the substrate.

The characteristic impedance, usually 50 or 75 Ω, is a very important parameter of the transmission line. Impedance matching between the transmission line, the device, and the measurement instrument will result in the most efficient signal transmission. For the common coplanar waveguide transmission line in the microwave field, its characteristic impedance can be calculated by its geometric parameters and relative dielectric constant of the substrate. According to the present research results, the characteristic impedance of the CPW transmission line shown in Figure 1 can be expressed as [45]

(1) $$Z_{0}=\frac{30\pi }{\sqrt{\varepsilon_{e}}}\frac{K(k)}{K(k^{\prime })}$$

where *ε_e_* is the effective dielectric constant of CPW transmission line, and can be expressed as

(2) $$\varepsilon_{e}=1+\frac{\varepsilon_{r}-1}{2}\frac{K(k^{\prime })}{K(k)}\frac{K(k_{1})}{K(k_{1}{}^{\prime })}$$

and

(3) $$\frac{K(k)}{K^{\prime }(k)}=\{\begin{array}{ll} {\left[\frac{1}{\pi }\ln\left(2\frac{1+\sqrt{k^{\prime }}}{1-\sqrt{k^{\prime }}}\right)\right]}^{-1} & 0\leq k\leq \frac{1}{\sqrt{2}}\\ \frac{1}{\pi }\ln\left(2\frac{1+\sqrt{k}}{1-\sqrt{k}}\right) & \frac{1}{\sqrt{2}}\leq k\leq 1 \end{array}$$

where $k=\frac{a}{b}$, $a=\frac{w}{2}$, $b=\frac{w}{2}+s$, $K^{\prime }(k)=K(k^{\prime })$, $k^{\prime }=\sqrt{1-k^{2}}$, $k_{1}=\frac{\sinh(\pi a/2h)}{\sinh(\pi b/2h)}$, and ${k^{\prime }}_{1}=\sqrt{1-k_{1}^{2}}$. Therefore, the characteristic impedance of the CPW transmission line can be obtained by Equations (1)–(3) once we obtain the CPW substrate material and structure.

In the practical applications, a reflection coefficient is typically used to describe the impedance match of the transmission line. According to transmission line theory, the reflection coefficient of the transmission line, Γ, can be expressed as

(4) $$\Gamma =\frac{Z_{L}-Z_{S}}{Z_{L}+Z_{S}}$$

where *Z_L_* is the impedance of the transmission line, and *Z_S_* is the internal impedance of the signal source or the test equipment. For the microwave circuit, the characteristic impedance of the transmission line equals the internal impedance of the signal source or the test equipment, and typically is 50 Ω.

In engineering use, the S parameter is used to represent the reflection coefficient. With the S parameter, Equation (4) can be written as

(5) $$S_{11}=20\log\left|\Gamma \right|=20\log\left|\frac{Z_{L}-Z_{S}}{Z_{L}+Z_{S}}\right|$$

where S_11_ is the reflection coefficient in dB.

### 2.2. The Real Transmission Line Impedance Considering Surface Roughness

**Figure 2:**
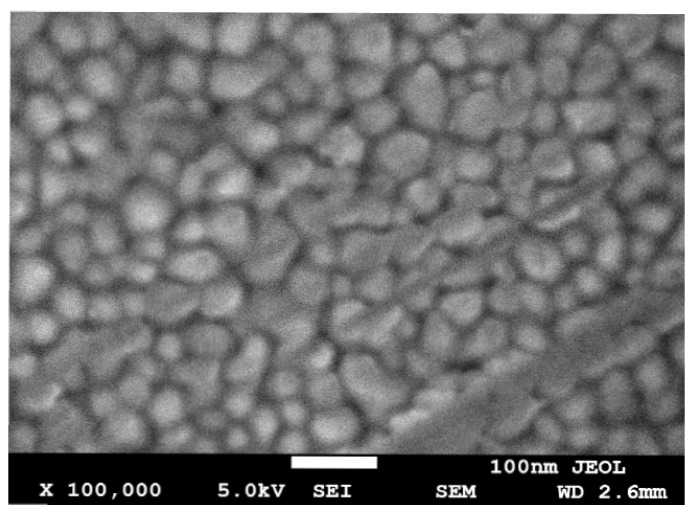
The SEM of a sputtered deposited Au film surface.

However, the aforementioned equations are based on the ideal model. Figure 2 is the surface of the scanning electron microscope (SEM) image of a sputtered deposited gold thin film surface. It can be seen that the surface of the Au thin film consists of many grains of the same size. When the grain size corresponds to the surface roughness of the device, especially in MEMS devices, the aforementioned equations need to be modified.

According to Maxwell’s equations [46],

(6) $$\{\begin{array}{l} {∯}_{S}E\cdot da=\frac{1}{\varepsilon_{0}}Q_{enc}\\ \;{∯}_{S}B\cdot da=0\\ \oint_{L}E\cdot dl=-\iint_{S}\frac{\partial B}{\partial t}\cdot da\\ \oint_{L}B\cdot dl=\mu_{0}\left(I_{0}+\varepsilon_{0}\iint_{S}\frac{\partial E}{\partial t}\cdot da\right) \end{array}$$

where is *S* the closed surface, d***a*** and d***l*** are the infinitesimal area and infinitesimal length, ***E*** is the electric field, ***B*** is the magnetic flux density, *ε*_0_ is the dielectric constant in vacuum, *L* is the closed curve, *μ*_0_ is the permeability of vacuum, *Q_enc_* is the electrical charge in the closed surface, and *I*_0_ is the current of the conduction current of the conductor.

**Figure 3:**
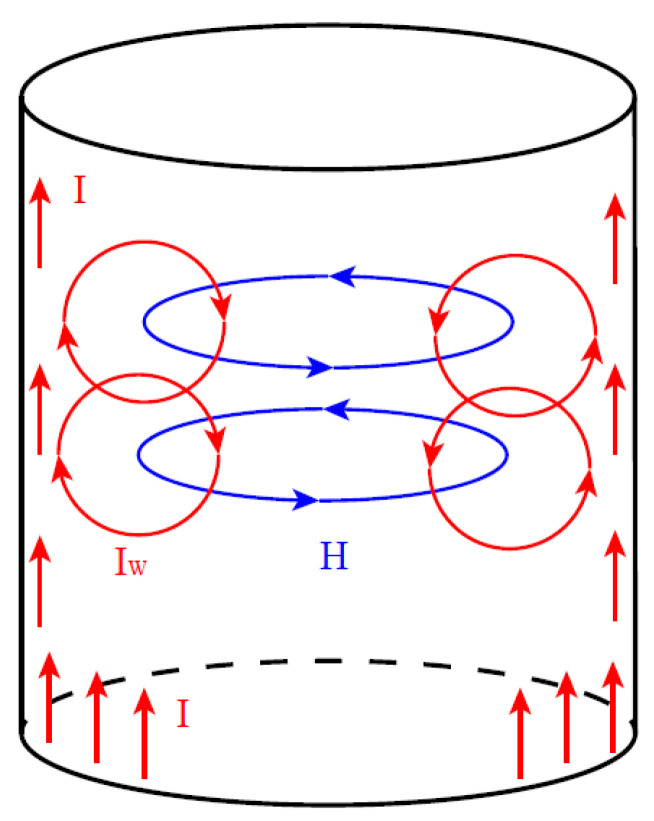
The illustration of the eddy current.

Figure 3 is the illustration of the eddy current. As shown in Figure 3, when a conductor is fed with a high-frequency alternating current I, a vortex magnetic field H is generated around the conductor current in the counterclockwise direction according to the fourth term of Equation (6). Accordingly, according to the third term of Equation (6), the vortex magnetic field generates a new vortex electric field, which in turn generates a vortex current (I_W_ in Figure 3), i.e., electromagnetic induction. The closer to the center of the conductor, the stronger the vortex electric field generated by electromagnetic induction. As shown in Figure 3, near the inside of the conductor, the newly generated vortex electric field cancels out with the original electric field; near the surface of the conductor, it superimposes with the original electric field, which leads to the concentration of current inside the metal conductor at the surface of the metal conductor.

**Figure 4:**
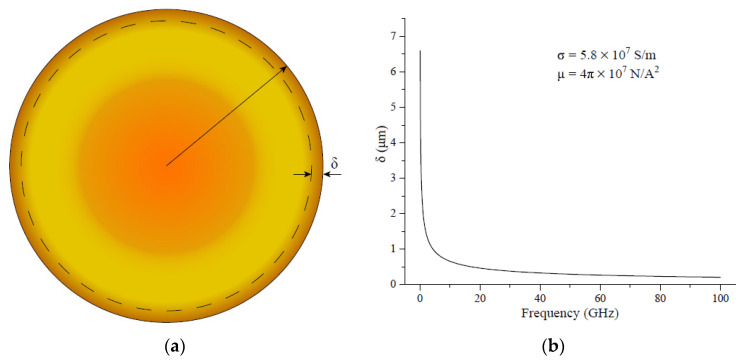
Skinning depth of the Cu: (**a**) skinning depth illustration; (**b**) skinning depth as frequency.

In high frequency circuits, the skinning depth is defined as the depth at which the current density decays to its surface value of 1/e (approximately 0.37). Therefore, the skinning depth can be expressed as

(7) $$\delta =\sqrt{\frac{2}{\omega \mu_{r}\mu_{0}\sigma }}$$

where *ω* is the frequency of the input current in rad/s, *μ_r_* is the relative permeability of the conductor, *μ*_0_ is the permeability of vacuum, and *σ* is the conductivity of the metal. Figure 4 shows the relationship between the skinning depth of Cu and the operating frequency. Figure 4a shows that the skin depth of Cu is a very thin portion of the metal surface and decreases rapidly with increasing operating frequency. When the frequency increases from 0.1 GHz to 100 GHz, the skin depth of Cu rapidly decreases from 6.8 μm to 0.4 μm. When the frequency is greater than 20 GHz, the skin depth of Cu metal is less than 0.5 μm, as shown in Figure 4b.

However, according to the current distribution for a rough surface conductor at high frequency in [42], the roughness peaks and valleys acted as discontinuities to current propagation, which increased the conductor resistance. Therefore, the impedance of the real transmission line is equaled to Equation (1) multiplied by a correction factor,

(8) $$Z_{rough}=Z_{0}r_{R}$$

where *r_R_* is defined as a rough factor. According the results of Groiss [46], it can be described by

(9) $$r_{R}=1+e^{-{\left(\frac{\delta }{2R_{q}}\right)}^{1.6}}$$

where *R_q_* is the RMS roughness of the conductor.

By defining the ratio of RMS roughness and skinning depth as a modified roughness coefficient, then the modified roughness coefficient can be expressed as

(10) $$r_{R}^{\delta }=\frac{\delta }{R_{q}}$$

Then, the rough factor in Equation (9) can be expressed as

(11) $$r_{R}=1+e^{-{\left(\frac{r_{R}^{\delta }}{2}\right)}^{1.2}}$$

Accordingly, the reflection coefficient of the actual transmission line can be expressed as

(12) $$\Gamma =\frac{Z_{rough}-Z_{s}}{Z_{rough}+Z_{s}}$$

If *Z_s_* = *Z*_0_, the reflection coefficient can be expressed as

(13) $$\Gamma =\frac{Z_{rough}-Z_{0}}{Z_{rough}+Z_{0}}$$

By substituting Equations (8) and (9) into Equation (13), we obtain that

(14) $$\Gamma =\frac{r_{R}Z_{0}-Z_{0}}{r_{R}Z_{0}+Z_{0}}=\frac{r_{R}-1}{r_{R}+1}=\frac{e^{-{\left(\frac{\delta }{2R_{q}}\right)}^{1.6}}}{2+e^{-{\left(\frac{\delta }{2R_{q}}\right)}^{1.6}}}\;$$

Then, S_11_ can be expressed as

(15) $$S_{11}=20\log|\Gamma |=20\log\left|\frac{e^{-{\left(\frac{\delta }{2R_{q}}\right)}^{1.6}}}{2+e^{-{\left(\frac{\delta }{2R_{q}}\right)}^{1.6}}}\right|$$

By substituting Equation (10) into Equations (14) and (15), we obtain that

(16) $$\Gamma =\frac{e^{-{\left(\frac{r_{R}^{\delta }}{2}\right)}^{1.6}}}{2+e^{-{\left(\frac{r_{R}^{\delta }}{2}\right)}^{1.6}}}$$

(17) $$S_{11}=20\log\left|\frac{e^{-{\left(\frac{r_{R}^{\delta }}{2}\right)}^{1.6}}}{2+e^{-{\left(\frac{r_{R}^{\delta }}{2}\right)}^{1.6}}}\right|$$

Therefore, we can use the modified roughness coefficient to analyze the electrical performances of the CPW transmission line.

## 3. Results and Discussion

**Figure 5:**
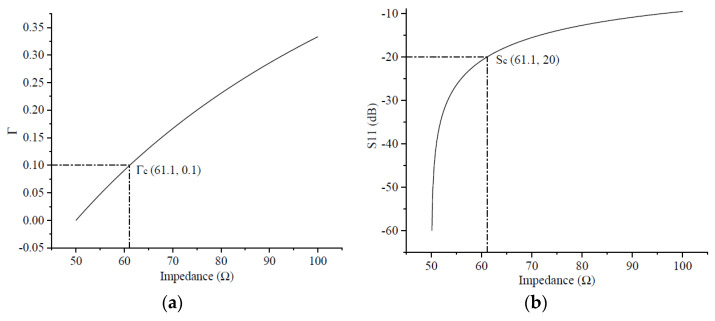
Relationship between reflection coefficient and impedance of the transmission line: (**a**) without units; (**b**) in dB.

In practical engineering applications, the reflection factor of a CPW transmission line is generally required to be no less than 20 dB in absolute value. This translates into a dimensionless constant of 0.1, meaning that only 10% of the input signal is allowed to be reflected. Substituting 0.1 into Equation (13), a simple calculation shows that the CPW transmission line impedance is about 61.1 Ω. For the sake of description, 61.1 Ω is considered the CPW transmission line threshold impedance, and 20 dB is considered the CPW transmission line threshold reflection coefficient, as shown in Figure 5 for Γc and Sc. After the impedance value was greater than the threshold impedance, the CPW transmission line was considered to have an impedance mismatch with the signal source or test equipment, and the transmission line could not be used at that frequency. The next step was to analyze the theoretical results obtained in Section 2 by means of a specific model.

**Figure 6:**
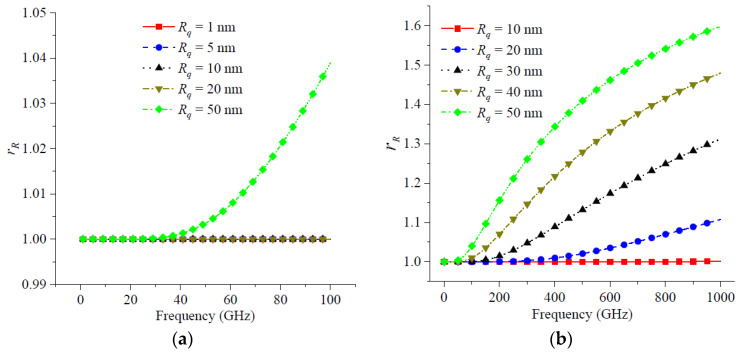
The rough factor and operation frequency: (**a**) the maximum frequency was 100 GHz; (**b**) the maximum frequency was 1000 GHz.

Figure 6 shows the roughness factors for different RMS surface roughness and the roughness factor at different operating frequencies. From Figure 6a, when the RMS surface roughness *R_q_* was less than 50 nm, the roughness factor *r_R_* did not increase with frequency in the frequency range of 1 GHz to 100 GHz. When the RMS surface roughness *R_q_* was equal to 50 nm, the roughness factor *r_R_* increased approximately in the frequency range from 1 GHz to 100 GHz. As the roughness factor measured the transmission line impedance, Figure 6a shows that for the transmission lines operating at 100 GHz, the RMS surface roughness had a limited effect on the impedance when the RMS roughness is less than 50 nm. Curran et al. [42] pointed out that the measured resistance of a transmission line with surface roughness was over 1.2 times to that of an ideal smooth transmission line. Gold et al. [28] showed that the insertion loss of a transmission line with RMS surface roughness of 1 μm at 65 GHz was about 3 times that of an ideal smooth transmission line. Therefore, higher surface roughness led to more significant effects on the transmission line performance.

However, current microelectronic devices are developing towards the very high frequency and even far-infrared frequency domain. With the continuous progress in the fabrication process, microelectronic devices will be applied to the ultra-high frequency and even far-infrared frequency domain in the future. In fact, there were already some applications of microelectronic devices in the far-infrared domain [1,2]. Therefore, the roughness factor beyond 100 GHz was considered in the next content.

From Figure 6b, when the operating frequency varied from 1 GHz to 1000 GHz, a decrease in the RMS surface roughness also led to a large change in the roughness factor. For example, the roughness factor increased by 0.6 when *R_q_* = 50 nm for a frequency range of 1 GHz to 1000 GHz, as shown in Figure 6b. In addition, the roughness factor increased by 0.1 when *R_q_* = 20 nm for a frequency range of 1 GHz to 1000 GHz. This indicated that the RMS surface roughness had a significant effect on the device performance at ultra-high frequencies and in the far-infrared frequency domain.

**Figure 7:**
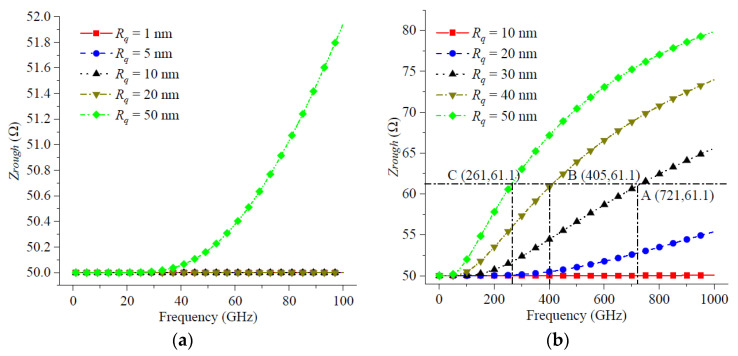
The impedance of transmission line and operation frequency: (**a**) the maximum frequency is 100 GHz; (**b**) the maximum frequency is 1000 GHz.

Next, the surface roughness at high frequencies was studied from the perspective of CPW transmission line impedance. As shown in Figure 7a, when the RMS surface roughness *R_q_* was less than 50 nm, the CPW transmission line impedance *Z_rough_* basically does not change with the increase of operating frequency in the frequency range from 1 GHz to 100 GHz. As *R_q_* increased to 50 nm, the transmission line impedance *Z_rough_* increased by approximately 2 Ω over the frequency range from 1 GHz to 100 GHz. This was different from the previously proposed transmission line threshold impedance of 61.1 Ω. Therefore, the impedance mismatch caused by nanoscale surface roughness was basically not considered in the 100 GHz frequency range.

As shown in Figure 7b, the increase in transmission line impedance due to the RMS surface roughness became particularly pronounced when the operating frequency increased to 1000 GHz. From Figure 7b, it can be seen that the transmission line impedance increased to its threshold impedance of 61.1 Ω at 721 GHz for RMS surface roughness *R_q_* = 30 nm, which is shown at point A (721, 61.1). At the same time, the operating frequency at which the transmission line threshold impedance was reached decreased rapidly with increasing *R_q_*. At *R_q_* = 40 nm, the threshold frequency decreased to 405 GHz, as shown in point B (405, 61.1) in Figure 7b. When *R_q_* = 50 nm, the threshold frequency decreased to 261 GHz, which was shown at point C (261, 61.1) in Figure 7b. Figure 7 showed that as the transmission line RMS surface roughness increased, the transmission line operating band became narrower in order not to affect its transmission performance.

**Figure 8:**
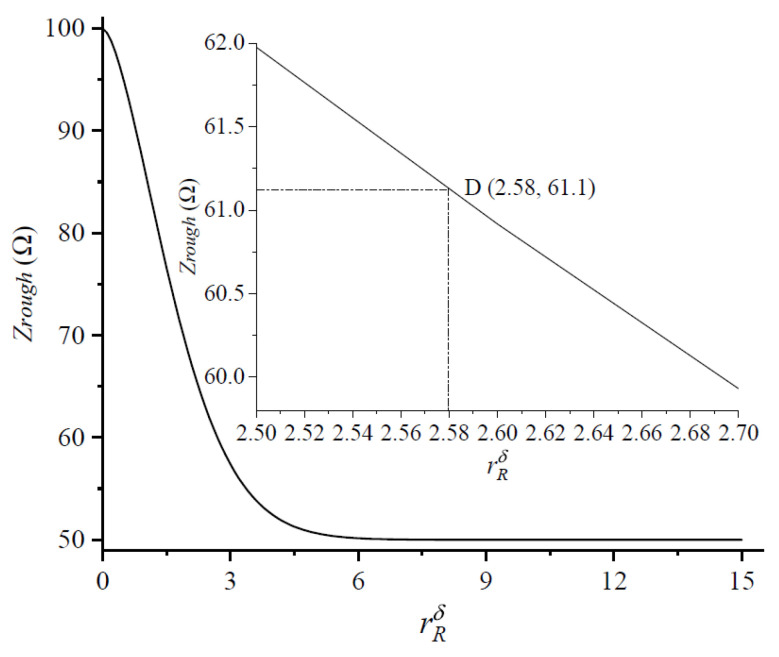
Modified rough coefficient and impedance of CPW transmission line.

The correspondence between the modified roughness coefficient and the transmission line impedance is shown in Figure 8. After a simple calculation, the modified roughness coefficient was 2.58 when the threshold impedance was reached, which was defined as the threshold modified roughness coefficient, as shown at point D (2.58, 61.1) in Figure 8. It can be seen that the transmission line impedance increased as the modified roughness coefficient decreased. The threshold modified roughness coefficient, like the transmission line threshold impedance, was a measure of the reflection rate of the transmission line signal up to 10%. As can be seen in Figure 8, the use of a roughness factor simplified the formulation of the roughness problem. An impedance mismatch between the transmission line and the signal source occurred regardless of the RMS surface roughness of the transmission line and the operating frequency, if the roughness factor was less than 2.58.

Because the reflection coefficient is more widely used in practical engineering applications, next, the problems related to nanoscale surface roughness were investigated from the perspective of transmission line reflection coefficient.

**Figure 9:**
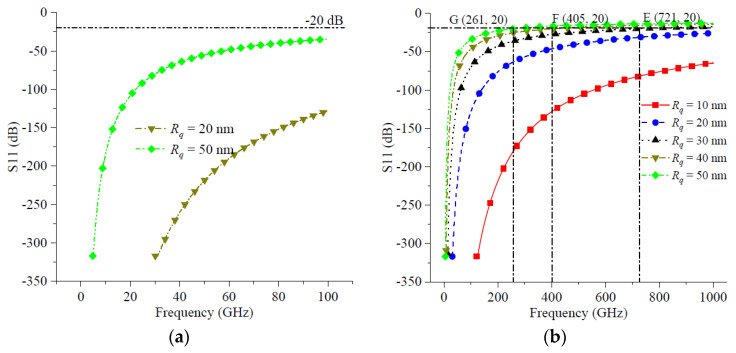
The reflection coefficient and operation frequency: (**a**) maximum frequency was 100 GHz; (**b**) maximum frequency was 1000 GHz.

As shown in Figure 9a, the absolute value of the reflection coefficient was less than the threshold reflection coefficient by 20 dB over the entire operating frequency range from 1 GHz to 100 GHz when the RMS surface roughness *R_q_* was not greater than 50 nm. Figure 9a shows that the RMS surface roughness had little effect on the reflection coefficient of the transmission line when the operating frequency was below 100 GHz.

As shown in Figure 9b, the effect of RMS surface roughness on the reflection coefficient started to be significant when the operating frequency increased to 1000 GHz. At this point, the threshold reflection coefficient could be reached at a small RMS surface roughness, and the operating frequency at which the threshold reflection coefficient was reached decreased rapidly with the increase in *R_q_*. When *R_q_* = 30 nm, the operating frequency at the threshold reflection coefficient was 721 GHz, which is shown at point E (721, 20) in Figure 9b. When *R_q_* increases to 40 nm, the operating frequency decreased to 405 GHz when the threshold reflection coefficient was reached, which is shown at point F (405, 20) in Figure 9b. As *R_q_* increased to 50 nm, the operating frequency at the threshold reflection coefficient decreased rapidly to 261 GHz, as shown at point G (261, 20) in Figure 9b.

Figure 9 shows that when the operating frequency was below 100 GHz, the effect of roughness on the transmission line reflection coefficient was very small and could be ignored. However, when the operating frequency increased to or over 100 GHz, the lower surface roughness also caused the degradation of the transmission line performance.

**Figure 10:**
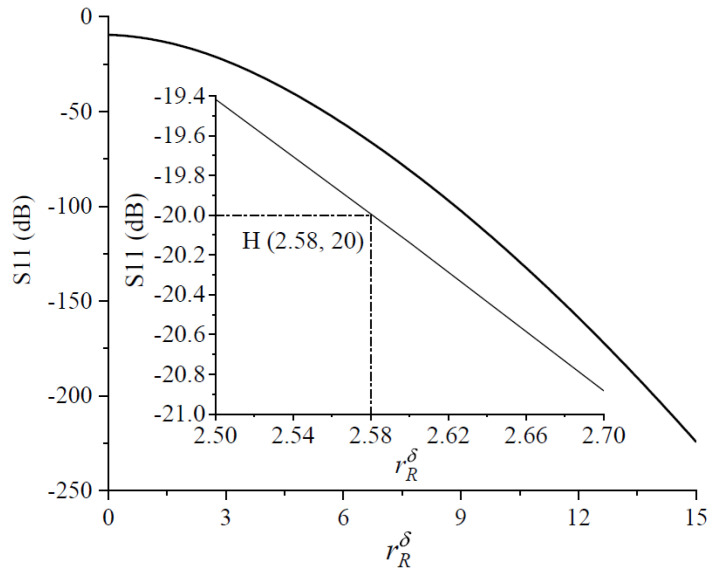
S11 and rough coefficient.

Next, the relationship between the modified roughness coefficient and the reflection coefficient was analyzed. As shown in Figure 10, the transmission line reached the threshold reflection coefficient when the modified roughness coefficient was 2.58, which is shown at point H (2.58, 20) in Figure 10. As the modified roughness coefficient decreased, the absolute value of the reflection coefficient decreased rapidly.

Figure 10 showed that, regardless of RMS surface roughness of the transmission line and the operating frequency, if the modified roughness coefficient was less than or equal to 2.58, the electrical performance of the CPW transmission line degraded. Compared with the correlation factor proposed in [8,9,10,11,12], the modified roughness coefficient proposed in this paper could be a critical value to determine the effect of surface roughness and work frequency on the S parameters of the transmission line.

## 4. Conclusions

This paper proposed a threshold impedance, a threshold reflection coefficient, and a modified roughness coefficient for the effect of surface roughness on transmission line transmission performance. The modified roughness coefficient considered the effects of surface roughness and skinning depth. The effects of RMS surface roughness and modified roughness coefficient on transmission line impedance and reflection coefficient were analyzed. The following conclusions could be drawn:

(1) When the transmission line was operated in the frequency range of 100 GHz and the RMS surface roughness was less than 50 nm, the effect of surface roughness on the transmission line impedance and reflection coefficient was limited and basically negligible.

(2) When the transmission line worked at very high frequency and in even the far-infrared frequency domain, the influence of RMS surface roughness on its impedance and reflection coefficient should be considered. The larger RMS surface roughness had a more significant influence of the transmission line transmission performance.

(3) The maximum operating frequencies of the transmission lines for different RMS surface roughness were given.

(4) Regardless of the RMS surface roughness and operating frequency, if the modified roughness coefficient was less than 2.58, an impedance mismatch occurred in the transmission line.

(5) The modified roughness coefficient proposed in this paper described the relationship between RMS surface roughness and transmission line impedance more clearly at high frequencies.

With the current microelectronic devices operating at higher and higher frequencies, transmission lines will also be used in the future for extremely high frequencies and even in the far-infrared frequency domain. At that time, the surface roughness problem will become an important issue for engineers. The method proposed in this paper can be used as a guideline for engineers to quickly measure whether to consider the nanoscale surface roughness problem or not, which is of certain guidance for engineering practice.

## Data Availability

Not applicable.

## References

[1] Talai A., Steinhäußer F., Gmeiner B., Wegener M., Bittner A., Deisinger U., Schmid U., Roosen A., Weigel R., Koelpin A. Electromagnetic analysis of conductor track surface roughnesses from 1 GHz to 110 GHz. Proceedings of the 2014 International Conference on Electromagnetics in Advanced Applications (ICEAA).

[2] Lau I., Hajian A., Michler F., Gold G., Lurz F., Schmid U., Helmreich K., Weigel R., Koelpin A. (2019). Influence of the PCB manufacturing process on the measurement error of planar relative permittivity sensors up to 100 GHz. IEEE Trans. Theory Tech..

[3] Hall S.H., Hall G.W., McCall J.A. (2000). High Speed Digital System and Design.

[4] Morgan S.P. (1949). Effect of surface roughness on eddy current losses at microwave frequencies. J. Appl. Phys..

[5] Chen C.-D., Tzuang C.-K.C., Peng S.T. (1992). Full-wave analysis of a lossy rectangular waveguide containing rough inner surfaces. IEEE Microw. Guided Wave Lett..

[6] Levie R.D. (1965). The influence of surface roughness of solid electrodes on electrochemical measurements. Electrochim. Acta.

[7] Chen X. (2007). EM modeling of microstrip conductor losses including surface roughness effect. IEEE Microw. Wirel. Compon. Lett..

[8] Sijercic E., Pejcinovic B. Modeling surface roughness with an array of hemispheres. Proceedings of the 33rd International Convention MIPRO.

[9] Hall S., Pytel S.G., Huray P.G., Hua D., Moonshiram A., Brist G.A., Sijercic E. (2007). Multigigahertz causal transmission line modeling methodology using a 3-D hemispherical surface roughness approach. IEEE Trans. Theory Tech..

[10] Huray P.G., Oluwafemi O., Loyer J., Bogatin E., Ye X. Impact of copper surface texture on loss: A model that works. Proceedings of the DesignCon 2010.

[11] Huray P.G., Hall S., Pytel S., Oluwafemi F., Mellitz R., Hua D., Ye P. (2020). Fundamentals of a 3-D snowball model for surface roughness power losses. IEEE Electromagn. Compat. Mag..

[12] Pérez-Arancibia C., Zhang P., Bruno O.P., Lau Y.Y. (2014). Electromagnetic power absorption due to bumps and trenches on flat surfaces. J. Appl. Phys..

[13] Yi M., Li S., Yu H., Khan W., Ulusoy C., Vera-Lopez A., Papapolymerou J., Swaminathan M. (2016). Surface roughness modeling of substrate integrated waveguide in D-band. IEEE Trans. Theory Tech..

[14] Guo X., Jackson D.R., Koledintseva M.Y., Hinaga S., Drewniak J.L., Chen J. (2014). An analysis of conductor surface roughness effects on signal propagation for stripline interconnects. IEEE Trans. Electromagn. Compat..

[15] Zhang L., Deng H., Zhao Y., Jiang W., Ning Y. Propagation characteristics of rectangular waveguide with fractal-regular rough surfaces. Proceedings of the 2009 9th International Conference on Electronic Measurement & Instruments.

[16] Kumar S., Sharma R. (2018). Analytical model for resistivity and mean free path in on-chip interconnects with rough surfaces. IEEE Trans. Emerg. Top. Comput..

[17] Kumar S., Sharma R. (2017). Analytical modeling and performance benchmarking of on-chip interconnects with rough surfaces. IEEE Trans. Multi-Scale Comput. Syst..

[18] Li N., Zheng F. (2011). Effect of micro/nano-scale rough surface on power dissipation of the waveguide: Model and simulate. J. Nanosci. Nanotechnol..

[19] Li N., Zheng F. Effect of micro nano-scale rough surface on the quality factor of the filter model and simulation. Proceedings of the 2010 IEEE International Conference on Mechatronics and Automation.

[20] Curran B., Fotheringham G., Tschoban C., Ndip I., Lang K.-D. (2016). On the modeling, characterization, and analysis of the current distribution in PCB transmission lines with surface finishes. IEEE Trans. Theory Tech..

[21] Manfredi P., Vande Ginste D., De Zutter D. (2015). An effective modeling framework for the analysis of interconnects subject to line-edge roughness. IEEE Microw. Wirel. Compon. Lett..

[22] Sain A., Melde K.L. (2013). Broadband characterization of coplanar waveguide interconnects with rough conductor surfaces. IEEE Trans. Comp. Pack. Manuf. Technol..

[23] Leung T., Xiaoxiong G., Braunisch H. (2006). Effects of random rough surface on absorption by conductors at microwave frequencies. IEEE Microw. Wirel. Compon. Lett..

[24] Ehsan M.A., Zhou Z., Liu L., Yi Y. (2015). An analytical through silicon via (TSV) surface roughness model applied to a millimeter wave 3-D IC. IEEE Trans. Electromagn. Compat..

[25] Gu X., Tsang L., Braunisch H., Xu P. (2007). Modeling absorption of rough interface between dielectric and conductive medium. Microw. Opt. Technol. Lett..

[26] Katayama K., Tanaka K., Tanaka M. (2022). Numerical analysis of frequency characteristics of transmitted waves by random waveguide. AIP Adv..

[27] Katayama K., Tanaka K., Tanaka M. (2021). Numerical simulations of resonant electromagnetic fields in the waveguide with random shape boundary. IEEE Trans. Theory Tech..

[28] Gold G., Helmreich K. (2017). A physical surface roughness model and its applications. IEEE Trans. Theory Tech..

[29] Gold G., Lomakin K., Helmreich K., Arz U. (2019). High-frequency modeling of coplanar waveguides including surface roughness. Adv. Radio Sci..

[30] Chen L., Tang M., Mao J. (2018). A semianalytical gradient model for characterization of conductors with surface roughness. IEEE Trans. Theory Tech..

[31] Huang B., Jia Q. (2019). Accurate modeling of conductor rough surfaces in waveguide devices. Electronics.

[32] Abdolhamidi M., Mohammad-Taheri M. (2019). Rough conductor modeling through state-space formulation. IEEE Trans. Theory Tech..

[33] Lomakin K., Gold G., Helmreich K. (2018). Analytical waveguide model precisely predicting loss and delay including surface roughness. IEEE Trans. Theory Tech..

[34] Grujic D.N. (2022). Simple and accurate approximation of rough conductor surface impedance. IEEE Trans. Theory Tech..

[35] Ding R., Tsang L., Braunisch H., Chang W. (2012). Wave propagation in parallel plate metallic waveguide with finite conductivity and three dimensional roughness. IEEE Trans. Antenn. Propag..

[36] Sanamzadeh M., Tsang L., Johnson J.T. (2019). 3-D Electromagnetic scattering from multilayer dielectric media with 2-D random rough interfaces using T-matrix approach. IEEE Trans. Antenn. Propag..

[37] Chen Q., Wong N. An efficient stochastic integral equation method for modeling the influence of conductor surface roughness on interconnect ohmic loss. Proceedings of the 2007 50th Midwest Symposium on Circuits and Systems.

[38] Gu X., Tsang L., Braunisch H. (2007). Estimation of roughness-induced power absorption from measured surface profile data. IEEE Microw. Wirel. Compon. Lett..

[39] Ding R., Tsang L., Braunisch H. (2012). Random rough surface effects in waveguides using mode matching technique and the method of moments. IEEE Trans. Comp. Pack. Manuf. Technol..

[40] Hammerstad E., Jensen O. Accurate models for microstrip computer-aided design. Proceedings of the 1980 IEEE MTT-S International Microwave symposium Digest.

[41] Ma X., Ochoa J.S., Cangellaris A.C. A method for modeling the impact of conductor surface roughness on waveguiding properties of interconnects. Proceedings of the 2013 IEEE 22nd Conference on Electrical Performance of Electronic Packaging and Systems.

[42] Curran B., Ndip I., Guttowski S., Reichl H. (2010). A methodology for combined modeling of skin, proximity, edge, and surface roughness effects. IEEE Trans. Theory Tech..

[43] Akinwale F., Engin A.E. (2011). A novel approach to the measurement and characterization of losses due to surface roughness in high speed transmission lines. Addit. Pap. Present..

[44] Ghione G., Naldi C. (1984). Analytical formulas for coplanar lines in hybrid and monolithic MICs. Electron. Lett..

[45] Groiss S., Bardi I., Biro O., Preis K., Richter K.R. (1996). Parameters of lossy cavity resonators calculated by the finite element method. IEEE Trans. Magn..

[46] Monk P. (2003). Finite Element Methods for Maxwell’s Equations.

